# Redefining the Role of Ornithine Aspartate and Vitamin E in Metabolic-Dysfunction-Associated Steatotic Liver Disease through Its Biochemical Properties

**DOI:** 10.3390/ijms25136839

**Published:** 2024-06-21

**Authors:** Larisse Longo, Rafael Aguiar Marschner, Laura Bainy Rodrigues de Freitas, Laura Renata de Bona, Luiza Behrens, Matheus Henrique Mariano Pereira, Valessa Emanoele Gabriel de Souza, Luiza Cecília Leonhard, Giulianna Zanettini, Carlos Eduardo Pinzon, Guilherme Jorge Semmelmann Pereira Lima, Carlos Thadeu Schmidt Cerski, Carolina Uribe-Cruz, Simone Magagnin Wajner, Mário Reis Álvares-da-Silva

**Affiliations:** 1 Graduate Program in Gastroenterology and Hepatology, Universidade Federal do Rio Grande do Sul, Porto Alegre 90010-150, Rio Grande do Sul, Brazil; llongo@hcpa.edu.br (L.L.); laurabrfreitas@gmail.com (L.B.R.d.F.); lbona@hcpa.edu.br (L.R.d.B.); ccerski@hcpa.edu.br (C.T.S.C.); investigacion1@ucami.edu.ar (C.U.-C.); mrsilva@hcpa.edu.br (M.R.Á.-d.-S.); 2 Experimental Laboratory of Hepatology and Gastroenterology, Center for Experimental Research, Hospital de Clínicas de Porto Alegre, Porto Alegre 90035-903, Rio Grande do Sul, Brazil; behrens.luiza@gmail.com (L.B.); mdmatheuspereira@gmail.com (M.H.M.P.); valessagabriel1993@gmail.com (V.E.G.d.S.); luiza_leonhardt@icloud.com (L.C.L.); giullyzanettini@gmail.com (G.Z.); carlos27pinsson@hotmail.com (C.E.P.); guilhermepl14@gmail.com (G.J.S.P.L.); 3 Endocrine Division, Hospital de Clínicas de Porto Alegre, Universidade Federal do Rio Grande do Sul, Porto Alegre 90035-903, Rio Grande do Sul, Brazil; rmarschner@hcpa.edu.br; 4 Unit of Surgical Pathology, Hospital de Clínicas de Porto Alegre, Porto Alegre 90035-903, Rio Grande do Sul, Brazil; 5 Facultad de Ciencias de la Salud, Universidad Católica de las Misiones, Posadas 3300, Misiones, Argentina

**Keywords:** animal model, metabolic-dysfunction-associated steatotic liver disease, ornithine aspartate, oxidative stress, steatohepatitis, type 3 deiodinase, vitamin E

## Abstract

It is known that the inflammation process leading to oxidative stress and thyroid hormone metabolism dysfunction is highly altered in metabolic dysfunction associated with steatotic liver disease (MASLD). This study aims to address the effect of ornithine aspartate (LOLA) and vitamin E (VitE) in improving these processes. Adult Sprague-Dawley rats were assigned to five groups and treated for 28 weeks: controls (*n* = 10) received a standard diet (for 28 weeks) plus gavage with distilled water (DW) from weeks 16 to 28. MASLD groups received a high-fat and choline-deficient diet for 28 weeks (MASLD group) and daily gavage with 200 mg/kg/day of LOLA, or twice a week with 150 mg of VitE from weeks 16–28. LOLA diminished collagen deposition (*p* = 0.006). The same treatment diminished carbonyl, TBARS, and sulfhydryl levels and GPx activity (*p* < 0.001). Type 3 deiodinase increased in the MASLD group, downregulating T3-controlled genes, which was corrected in the presence of LOLA. LOLA also promoted a near-normalization of complex II, SDH, and GDH activities (*p* < 0.001) and improved reticulum stress, with a reduction in GRP78 and HSPA9/GRP75 protein levels (*p* < 0.05). The enhanced energy production and metabolism of thyroid hormones, probably because of GSH replenishment provided by the L-glutamate portion of LOLA, opens a new therapeutic approach for MASLD.

## 1. Introduction

In Western countries, the prevalence of metabolic dysfunction associated with steatotic liver disease (MASLD) ranges from 30% to 40% within the general population. However, this condition affects up to one-third of individuals residing in South America or the Middle East [1,2,3]. Presently, cirrhosis resulting from steatohepatitis is a prominent cause of liver-related mortality [4]. The precise mechanisms contributing to the development and progression of MASLD still need to be discovered. However, numerous factors act concomitantly, including genetic variants, aberrant lipid metabolism, oxidative stress, perturbed immune response, hormonal alterations, and imbalances in the composition of the gut microbiota, among others [5,6,7]. Recently, studies have revealed the involvement of thyroid metabolism (TM) in the liver and the significance of alterations in thyroid hormones (TH) in the progression of MASLD. This mechanistic understanding has sparked interest in exploring novel therapeutic approaches that target this pathway [8,9]. Resmetirom, a TH receptor beta-selective agonist, has just become the first approved agent for treating MASLD. However, given the multifactorial nature of the disease, there is a growing need to explore more comprehensive treatment approaches.

A putative mechanism for L-ornithine-L-aspartate (LOLA) was recently elegantly proposed [10]. In this animal model, the resulting reduced glutathione (GSH) production would be able to replenish the antioxidant reserves of the cells. At the same time, the produced glutamine would work through the urea pathway. Hepatic alterations in metabolism, catabolism, and the ammonia detoxification process are potential targets for treating MASLD [10,11]. Therefore, LOLA could help treat the disease due to the production of glutamate [10], which may contribute to the amelioration of several abnormal metabolic steps. Additionally, other studies recommended Vitamin E (VitE), a lipid-soluble chain-breaking antioxidant, as an additional treatment [12,13,14].

In this study, we aimed to assess the effects of LOLA and VitE on the progression of liver inflammation and fibrosis in a steatohepatitis experimental model. Specifically, we investigated the impact of these interventions on oxidative stress, Krebs cycle enzymes, endoplasmic reticulum stress, and thyroid hormone metabolism.

## 2. Results

### 2.1. General Characteristics

The baseline body weights of the animals in all the experimental groups were similar (*p* = 0.999). At the end of the experimental period, the animals in the MASLD, LOLA, and VitE experimental groups showed a significant increase (*p* < 0.001) in their abdominal circumference, abdominal adipose tissue accumulation, fresh liver weight, and liver weight/body weight ratio compared with those in the control group.

### 2.2. Biochemical Parameters

As expected, animals in the MASLD group had higher serum levels of ALT (*p* < 0.01), glucose (*p* < 0.05), TC (*p* = 0.03), and LDL cholesterol (*p* = 0.009), with lower levels of HDL cholesterol (*p <* 0.001). Serum ALT levels and triglycerides were significantly lower in animals receiving therapeutic intervention than in the MASLD group (*p* < 0.01 and *p* < 0.005, respectively). The data obtained are shown in Table 1.

### 2.3. Expression of Genes Involved in Steatohepatitis Pathogenesis

The results obtained for the gene expression of hepatic inflammatory markers are reported in Table 1. The inflammatory markers showed a significant increase in the levels of *Il1b* (*p* < 0.05) and *Il6* (*p* < 0.05) in the MASLD group compared with the control group. Expression of *Il10* was significantly higher in animals treated with LOLA than in those that received VitE (*p* < 0.05).

### 2.4. Concentration of Soluble Vascular Adhesion Protein-1

There was a significant increase in the serum concentration of VAP-1 in the MASLD group compared with that in the control group (*p* < 0.05). No substantial reduction in this marker was observed in animals treated with LOLA or Vit E (Table 1).

### 2.5. Analysis of Fat Deposition in Liver Tissue

**Figure 1:**
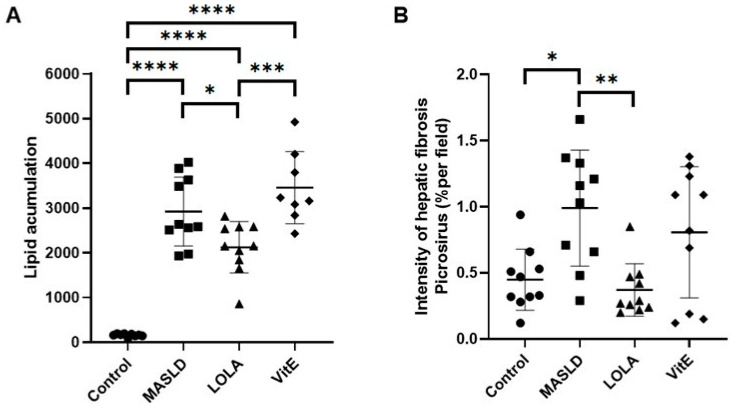
(**A**) Accumulation of liver lipids and (**B**) quantification of collagen fibers. Data expressed as mean ± standard deviation. Different letters indicate a significant difference between groups (*p* < 0.05). * Indicates a significant difference between groups: * *p* < 0.05, ** *p* < 0.01, *** *p* < 0.001, **** *p* < 0.0001. LOLA: ornithine aspartate; MASLD: metabolic dysfunction-associated fatty liver disease; and VitE: vitamin E.

The animals in the MASLD, LOLA, and VitE groups showed a significant increase in the accumulation of total lipids in the liver tissue compared with that in the control group (*p* = 0.003; Figure 1A). Among the groups that received treatment, there was a decrease in the accumulation of fat deposition in liver tissue in the LOLA group compared with the VitE group (*p* = 0.001).

### 2.6. Histopathological Analysis

No abnormalities were observed in the liver tissue of the control group animals. In contrast, the MASLD, LOLA, and VitE groups’ animals had predominant microvesicular steatosis, moderate macrovesicular steatosis, inflammatory activity, and mild hypertrophy. Regarding the quantification of collagen in the picrosirius-stained slides, there was a significant increase of 2.2% in the MASLD group compared with the control group (*p* = 0.009). The quantification of hepatic fibrosis, using picrosirius staining, was lower in the LOLA group when compared to MASLD animals (*p* = 0.002). VitE did not change the fibrosis process (Figure 1B).

### 2.7. Oxidative Stress and Antioxidative Defenses in Liver Tissue

**Figure 2:**
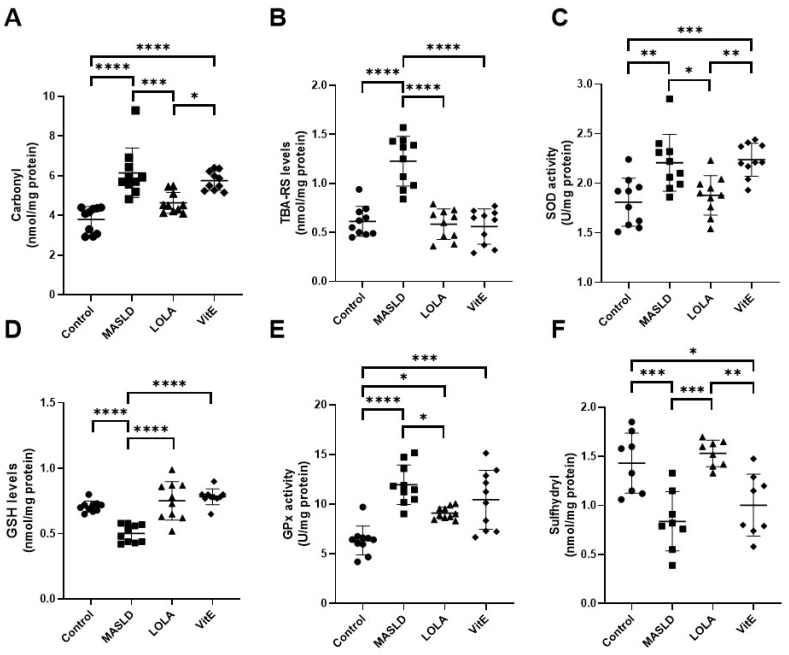
Oxidative stress parameters and antioxidative defenses: (**A**) carbonyl, (**B**) thiobarbituric acid-reactive substances (TBA-RS), (**C**) reduced glutathione (GSH), (**D**) glutathione peroxidase (GPx), (**E**) superoxide dismutase (SOD), and (**F**) sulfhydryl content. Data are expressed as the mean ± standard deviation. Asterisks indicate a significant difference between groups: * *p* < 0.05, ** *p* < 0.01, *** *p* < 0.001, **** *p* < 0.0001. LOLA: ornithine aspartate; MASLD: metabolic-dysfunction-associated fatty liver disease; VitE: vitamin E.

A significant increase in carbonyl formation, a marker of protein oxidation, was observed in the MASLD group (*p* < 0.001). Nevertheless, animals treated with LOLA showed a significant decrease in protein oxidation (*p* < 0.001; Figure 2A) comparable to that in the control group. The MASLD group showed a substantial increase in TBARS oxidative stress marker levels, normalizing its levels in the LOLA and VitE groups (*p* < 0.001 for all; Figure 2B).

As expected, MASLD showed a significant increase in SOD levels (*p* = 0.002) with VitE (*p* = 0.001; Figure 2C). The activity of this enzyme was similar between the LOLA and control groups (*p* = 0.905; Figure 2C). The levels of GSH, the liver’s most important antioxidant, were significantly lower in the MASLD group (*p* < 0.001). Interestingly, there was a significant increase in its levels in the groups treated with LOLA and VitE (*p* < 0.001; Figure 2D). There was a considerable increase in GPx activity in the MASLD group (*p* < 0.001), which was expected because of the association of GPx activity with GSH production. Decreased GPx activity in the LOLA group indicates that this compound improved the amount of substrate available for GSH production (Figure 2E) compared with the MASLD group. Sulfhydryl is a marker for the presence of thiols in the medium and is a substrate for GSH formation. Likewise, a significant increase in sulfhydryl levels was observed in the MASLD and VitE groups compared with the LOLA group (*p* = 0.021 and *p* = 0.035, respectively); alternatively, there was a reduction in the sulfhydryl levels in the group treated with LOLA compared with the MASLD group. This represents an increase in antioxidative defenses due to the production of GSH (Figure 2F).

### 2.8. Type 3 Deiodinase Measurement

**Figure 3:**
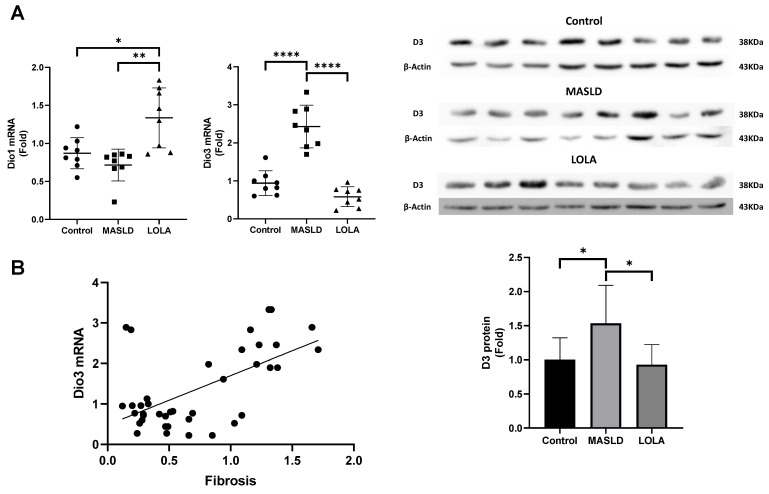
Assessment of thyroid metabolism in the liver. (**A**) *Dio1* and *Dio3* mRNA expression and D3 protein quantification. Expression of genes linked or stimulated by T3. (**B**) Correlation of D3 concentration with fibrosis in animals. Data are expressed as the mean ± standard deviation. Asterisks indicate a significant difference between groups: * *p* < 0.05, **p < 0.01, **** *p* < 0.0001. LOLA: ornithine aspartate; MASLD: metabolic-dysfunction-associated fatty liver disease.

Since no positive correction of the oxidative status was observed with VitE, we next evaluated only the effect of LOLA on the parameters susceptible to oxidative stress. Thus, Figure 3 displays the *Dio3* mRNA and protein levels that were increased in MASLD and regulated in the presence of LOLA (~25–30%; *p* < 0.0001; Figure 3A). With these findings, we correlated the concentration of D3 and the degree of fibrosis in the animals. We observed a positive correlation between the levels of D3 versus fibrosis (*p* < 0.001; Figure 3B).

### 2.9. Endoplasmic Reticulum Stress and Cell Apoptosis in Liver Tissue

**Figure 4:**
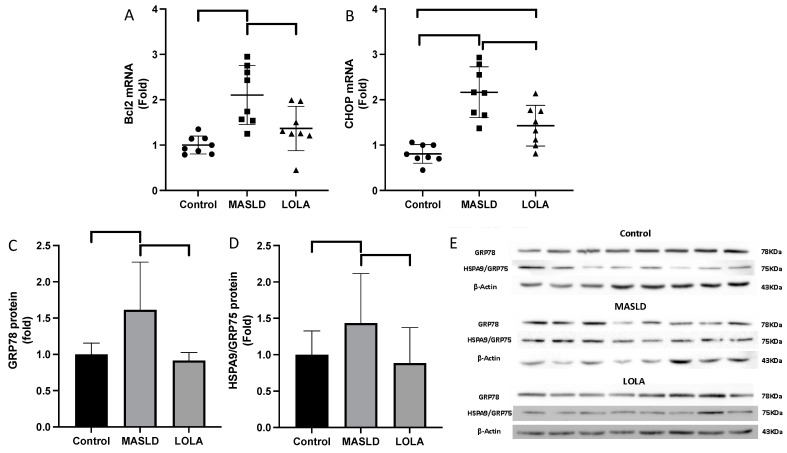
Apoptosis, energetic viability, and assessment of reticulum stress. (**A**) B-cell lymphoma 2 (*Bcl2*) mRNA expression. (**B**) C/EBP homologous protein *(Chop)* mRNA expression. (**C**) GRP78 protein quantification. (**D**) HSPA9/GRP75 protein quantification. (**E**) Western blot images and graphs of GRP78 and HSPA9/GRP75 in all groups. Data are expressed as the mean ± standard deviation. LOLA: ornithine aspartate; MASLD: metabolic-dysfunction-associated fatty liver disease.

Next, we proceeded to investigate the endoplasmic reticulum and cell viability. We observed an elevation in reticulum stress in the MASLD group, indicated by the increased expression of *Chop* mRNA and of GRP78 and HSPA9/GRP75 proteins (*p* < 0.05; Figure 4). However, this effect was reversed in the LOLA group (*p* < 0.001; Figure 4). When assessing cell viability, we observed an upregulation in the expression of *Bcl2* mRNA, an anti-apoptotic indicator, in the MASLD group. However, in the LOLA group, this effect was corrected (*p* < 0.001; Figure 4).

### 2.10. Krebs Cycle Enzymes in Liver Tissue

**Figure 5:**
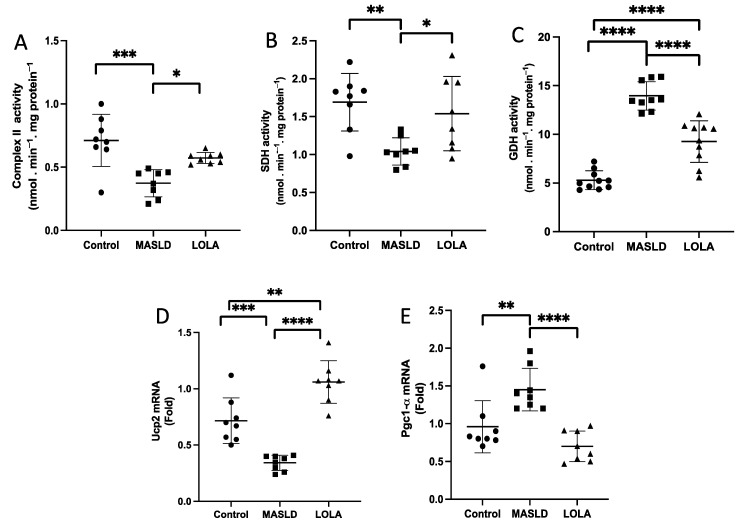
Krebs cycle markers and energy metabolism genes. (**A**) Complex II activity, (**B**) succinate dehydrogenase activity, (**C**) glutamate dehydrogenase (GDH) activity, and (**D**) uncoupling protein 2 (*Ucp2*) and (**E**) proliferator-activated receptor gamma coactivator-1α (*PGC1-α*) mRNA expression. Data are expressed as the mean ± standard deviation. Asterisks indicate a significant difference between groups: * *p* < 0.05, ** *p* < 0.01, *** *p* < 0.001, **** *p* < 0.0001. LOLA: ornithine aspartate; MASLD: metabolic-dysfunction-associated fatty liver disease.

Next, we sought to see whether the Krebs cycle was affected. We observed that although there was no change in complex II (Figure 5A), the activity of enzymes SDH, which demonstrates the capacity of the respiratory chain (Figure 5B), and GDH, which detoxifies ammonia (Figure 5C), were corrected by LOLA (*p* = 0.003). Regarding the energy production capacity, we noted decreased *UCP2* mRNA levels and an elevation in *PGC1-α* mRNA gene expression in the MASLD group (*p* < 0.001). In the LOLA-treated group, this expression was restored to levels comparable to those in the control group (*p* < 0.001; Figure 5D).

## 3. Discussion

In this study, we observed that animals with mild MASLD disease mimic the alterations corresponding to those reported in the initial stages of human disease. These results are probably secondary to the correction of GSH levels, and the produced ammonia goes back into the urea cycle since the glutamine levels are putatively corrected. Interestingly, LOLA could promote a reduction in the deposition of collagen fibers in liver tissue. Moreover, the type 3 deiodinase, which inactivates T3 and is augmented in disease, had its mRNA and protein levels normalized [5]. This is probably due to the correction of GSH and the sulfhydryl levels.

Interestingly, type 3 deiodinase levels directly correlated with fibrosis intensity. Regarding genes that T3 regulates, we observed that those responsible for mitochondrial health and endoplasmic reticulum stress were affected in MASLD and corrected in the presence of LOLA, probably secondary to T3 level correction. The SDH and GDH enzymes, which are part of the Krebs cycle and responsible for ammonia management, were better corrected with LOLA, suggesting that even in the initial disease, one can already observe the change in enzyme influx from the Krebs cycle and energy production to the urea cycle to detoxify ammonia.

A previous paper hypothesized that LOLA could effectively treat MASLD and steatohepatitis; however, further confirmation was necessary [10]. In this study, we demonstrated that animals subjected to HFCD presented a marked deposition of body and liver fat, with histopathological lesions characteristic of steatosis or steatohepatitis. Accordingly, the initial theory proved correct: improved GSH and glutamine levels improved the altered liver metabolism earlier in the disease than expected. These findings might be the most significant step toward understanding the role and timing of LOLA in hepatic disease, as correcting metabolic pathways might indicated at the diagnosis of the disease rather than when the damage is at a point of no return.

Decreasing and normalizing the biochemical parameters of the lipid profile is highly recommended in patients with MASLD, mainly because of the attendant risk of developing cardiovascular diseases [15]. Administration of LOLA promoted a better increase in the expression level of the anti-inflammatory *Il10* compared with all the other groups. This finding aligns with the observed redox correction, which diminishes the local inflammatory process. Additionally, the complete reorganization of the enzymatic profile of antioxidative molecules (SOD and GPx activity), plus the improvement in the amount of GSH, also helps to explain the accommodating inflammatory process. Along the same line, hepatic fibrosis was diminished in the presence of LOLA but not with VitE [16]. Again, inflammation and the constant activation of pro-inflammatory molecules that is interrupted in the presence of LOLA help explain such an important finding in stopping or delaying disease progression. In this context, the molecule VAP-1 can be a good marker of disease progression [17]. Corroborating the results obtained in this study, the increased serum concentration of VAP-1 is associated with obesity, diabetes, and inflammatory liver disease [17,18,19]. Of note, in patients with steatohepatitis, the serum VAP-1 levels correlate with the severity of obesity, steatohepatitis, and, more importantly, the fibrosis stage [20], but LOLA or VitE does not alter it.

The augmented D3 in MASLD has already been described in [5], leading to a whole cascade of alterations. This enzyme is known to be induced with inflammation and is a pro-oxidant in the illness [21,22,23,24]. Here, we demonstrate that the augmented D3 in MASLD liver is associated not only with mitochondrial damage, as shown by changes in the *Ucp2* and *Pgc1-α* genes but also with the induced *Bcl2* gene, which is not only an anti-apoptotic stimulus to the mitochondria but also acts as an oncogene that could link the whole process to a final hepatocellular carcinoma stage. The interaction between T3 and the mitochondria is intricate [25,26]. While *Ucp2* uncouples the organelle initiating the energy production process, *Bcl2* is anti-apoptotic, augmenting mitochondrial swelling, diminished energy production, and disarrangement of the tissue.

Moreover, *Chop*, GRP78, and HSPA9/Grp75 are endoplasmic reticulum stress markers. Together, these genes, controlled by T3, generate alterations that result in diminished energy production and gluconeogenesis and augment organelle dysfunction, resulting in a total disarrangement of the tissue. Another significant result described here is the alteration of the dehydrogenase enzymes of the Krebs cycle (dehydrogenase succinate and glutamate dehydrogenase), which are surprisingly altered at such an early stage of the disease. These most significant findings indicate augmented levels of these enzymes in MASLD despite diminished levels of the ion transport channel, complex II. Again, LOLA, through its mechanism of glutamine production, can decrease ammonia levels by increasing urea synthesis (L-ornithine is a metabolic intermediate in the urea cycle) by periportal hepatocytes and by increasing glutamine synthesis through the enzyme glutamine synthetase [10,27]. The results with SDH and GDH suggest that not only is the function of both enzymes corrected by LOLA but that these enzymes are also reoriented to the Krebs cycle and energy production instead of probably detoxifying ammonia. In an experimental model of liver failure resulting from toxic liver injury, treatment with LOLA was shown to correct the loss of GSH, thus corroborating the findings of our study [28].

Some limitations of this work deserve to be mentioned. We did not perform mitochondrial respiration in the fresh tissue, which would have added to the enzyme studies. Moreover, immunofluorescence assays could have added to the set of results showing the location of the proteins studied here. Nevertheless, these experiments will be performed in subsequent studies.

One of the most striking results of this research is the positive correlation between D3 and fibrosis. This correlation can extend the effect of LOLA in augmenting GSH levels and, thus, augmenting T3 levels in the tissue through D3 inhibition. Moreover, the pro-oxidative environment is amended, and the genes and enzymes responsible for energy production and tissue maintenance might be improved, thus improving an extremely unfavorable environment.

## 4. Materials and Methods

### 4.1. Animals and Ethical Procedures

Fifty adult (60-day-old) male Sprague-Dawley rats weighing 320–370 g were used. The animals were group-housed in polypropylene cages with sawdust-covered floors. Animals were maintained on a standard 12 h light/dark cycle in a temperature-controlled environment (22 ± 2 °C) with 40–60% humidity, and an exhaust system. The Institutional Ethics Committee approved all experiments and procedures for using animals (Protocol number: 2019-0297). The methods for using scientific animals were conducted as per the *Guide for the Care and Use of Laboratory Animals* (8th ed., 2011) and Law No. 11794 (Brazil, 2008).

### 4.2. Study Design

After the habituation period, all animals were randomized by weight into a control group (*n* = 10) that received a standard diet and four intervention groups (*n* = 40) that received a high-fat and choline-deficient (HFCD) diet. Each of the intervention groups received distilled water (MASLD group; *n* = 10), LOLA (LOLA group; *n* = 10), or VitE (VitE group; *n* = 10). Animals were weighed weekly during the experimental period. After 28 weeks of the experiment, all the animals were euthanized by cardiac exsanguination. Before euthanasia, the animals were fasted for eight hours. The liver was removed and weighed. Samples were frozen in liquid nitrogen and stored at −80 °C until the experimental procedures were carried out. A portion of each liver sample was fixed in 10% formalin for histological analysis.

### 4.3. Experimental Diets

Our research group developed the experimental model used in this study [6]. In summary, in accordance with a previous publication, the animals in the control group received a standard rodent diet (Nuvilab CR-1; Quimtia S.A., São Paulo, Brazil) with an energy value of 2.93 kcal/g. The diet consisted of 55.0% carbohydrates, 22.0% protein, and 4.5% fat, while 18.5% consisted of other nutrients such as fiber and vitamins. Animals in the intervention groups received an HFCD diet (RH19576; Rhoster, São Paulo, Brazil) with an energy value of 4.3 kcal/g. This product comprised 54.5% carbohydrates, 14.0% protein, and 31.5% fat (enriched with 54.0% trans-fatty acids) [6]. The diet of the intervention group mirrors many of the phenotypes observed in humans with MASLD, as previously demonstrated [6]. The diet offered to the animals in both groups was replaced every two days. Both groups received water and food ad libitum during the experimental period.

### 4.4. Ornithine Aspartate and Vitamin E Treatment

Previous information about the experimental model is described in previous studies [6,29]. However, in summary, after 16 weeks of the standard or HFCD diet, the therapeutic intervention was started daily with LOLA or VitE or vehicle (Veh) solution until euthanasia was performed [29]. Animals in the control and MASLD groups received daily gavage with a Veh solution (0.5 mL/kg distilled water). The LOLA group received a daily dose of 200 mg/kg (LOLA; Biolab Sanus Farmacêutica Ltd.a, Taboao Da Serra, Brazil), which alleviated liver damage in the MASLD [11,30,31]. The VitE group received a dose of 150 mg twice a week (VitE; Biolab Sanus Farmacêutica Ltd.a) and received gavage with Veh solution on the other days [13,32]. The administered dose of VitE can reduce inflammation, steatosis, and the deposition of collagen fibers mediated by reactive oxygen species, restoring insulin sensitivity [32].

### 4.5. Biochemical Analysis

The serum levels of aspartate aminotransferase (AST), alanine aminotransferase (ALT), glucose, total cholesterol (TC), low-density lipoprotein (LDL), high-density lipoprotein (HDL), and triglycerides were determined using Labmax 560 equipment (São Paulo, Brazil).

### 4.6. Real-Time PCR

Total RNA was extracted from tissues using the TRIzol method. A high-capacity cDNA reverse transcription kit (Applied Biosystems, Bedford, MA, USA) was used for cDNA conversion from 2 μg of RNA, followed by real-time PCR with SYBR Green PCR Power Up (Applied Biosystems) in an ABI Prism Vii7 Sequence Detection System (Applied Biosystems) assay. We analyzed the gene expression levels of interleukin-1b (Il1b), Il6, Il10, tumor necrosis factor-a (Tnfa), deiodinase type-1 (Dio1), Dio3, C/EBP homologous protein (Chop), B-cell lymphoma 2 (Bcl2), uncoupling protein 2 (Ucp2), proliferator-activated receptor gamma coactivator-1α (Pgc1a). The glyceraldehyde-3-phosphate dehydrogenase (Gapdh) and CyPA (cyclophilin-A) genes were used for normalization. The changes in gene expression levels were calculated using the formula 2^−(ΔΔCt)^. The primers used are listed in Table 2.

### 4.7. Histopathological Analysis

**Figure 6:**
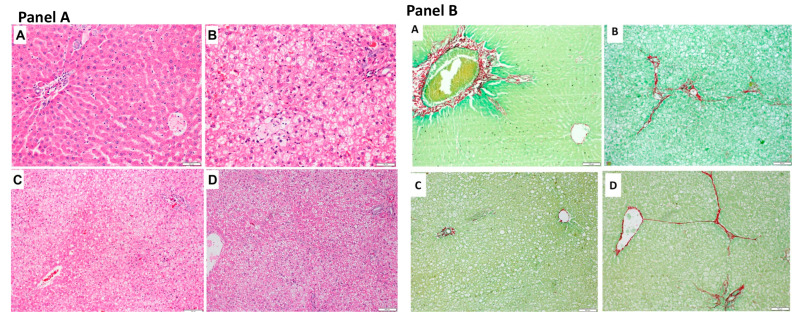
Liver histological evaluation. (**Panel A**): Images from the control (**A**), MASLD (**B**), LOLA (**C**), and VitE (**D**) groups. All samples in the images were stained with H&E and are shown at 40× magnification (**A**,**B**) or 20% magnification (**C**,**D**). (**Panel B**): Picrosirius-stained images from the control (**A**), MASLD (**B**), LOLA (**C**), and VitE (**D**) groups; all panels are shown at 20× magnification. LOLA: L-ornithine-L-aspartate; MASLD: metabolic-dysfunction-associated steatotic liver disease; VitE: vitamin E.

Formalin-fixed liver tissue samples were embedded in paraffin and stained with hematoxylin and eosin (H&E) and picrosirius red (Figure 6; Panel A). Histopathological lesions of the different evolutionary stages of NAFLD were assessed according to the score by Liang et al., a highly reproducible scoring system applicable to experimental rodent models [33]. An experienced pathologist, blinded to the experimental groups, performed the analysis. Fibrosis was quantified using morphometric analysis after picrosirius staining. Ten randomly selected field images were obtained per animal using the Olympus BX51 microscope, and the QCapture X64 program with 200× magnification was used to determine the staining intensity. This evaluation was performed using the ImageJ software (version 1.51p).

### 4.8. Quantitative Analysis of Liver Fat Deposition

The previously frozen liver tissue samples were thawed on ice and homogenized in phosphate buffer saline (20 mg of tissue/mL) to analyze the hepatic lipid content. Using this homogenate, the total lipid concentration was determined using the modified protocol of Gómez-Lechón et al. [34]. In short, liver tissue homogenized in a phosphate buffer was incubated with one μL of Nile Red solution (1 mg/mL in acetone) at 37 °C for 15 min. Fluorescence was measured at 488 nm excitation and 550 nm emission (SpectraMax M3, Invitrogen-Thermo Fischer, Waltham, MA, USA). The obtained values were normalized to the total protein present in the homogenate [35]. The results are expressed as fluorescence/μg protein and shown in Figure 6 (Panel B).

### 4.9. Soluble Vascular Adhesion Protein-1 Measurements

Serum levels of vascular adhesion protein-1 (VAP-1) were measured using an enzyme-linked immunosorbent assay (MyBioSource, Waltham, MA, USA). All analyses were performed in duplicate according to the manufacturer’s instructions. Absorbance was measured using a spectrophotometer at 450 nm (Zenyth 200 RT, New York, NY, USA). Concentrations were determined using a standard curve, and the results were expressed in ng/mL.

### 4.10. Oxidative Stress Parameters, Antioxidative Defenses, and Krebs Cycle Enzymes

#### 4.10.1. Carbonyl Measurement

The technique for assessing the carbonyl concentration was performed as described in [36]. Duplicate 0.3 mg aliquots of protein homogenates of each tissue were incubated with 500 μL of 10 mmol/L 2.4-dinitrophenylhydrazine or 1.0 mL of HCl 2 mol/L (blank tube). As previously described, 250 μL of trichloroacetic acid 50% v/v was added to the aliquots. The samples were centrifuged at 8000× *g* for 30 min to obtain the protein pellets, which were immediately washed with ethanol–ethyl acetate at a 1:1 v/v ratio. The difference between the 2.4-dinitrophenylhydrazine-treated and HCl-treated samples (blank) at 370 nm was used to calculate the carbonyl content. Carbonyl content was calculated using the millimolar absorption coefficient of hydrazine (e370 nm = 21,000,000 M^−1^ cm^−1^), and the results were expressed in nmol carbonyl/mg protein.

#### 4.10.2. Malondialdehyde (MDA) Levels

The technique for assessing MDA concentrations was performed as previously described [37]. The organic phase’s fluorescence was read at the wavelengths of 515 and 553 nm for excitation and emission, respectively. A calibration curve was performed with 1,1,3,3-tetra-methoxy propane that was subjected to the same treatment as the supernatant. MDA levels were calculated as nanomoles of MDA/mg protein.

#### 4.10.3. Sulfhydryl Content

Sulfhydryl content was measured accordingly [38], using the reduction of 5,5′-dithiol-bis (2-nitrobenzoic acid) (DTNB) by thiols to generate a yellow derivative (TNB) that is measured at 412 nm. In brief, 30 μL of DTNB 0.1 mmol/L was added to 120 μL of tissue supernatants. This was followed by a 30 min incubation at room temperature in a dark room. Results were calculated as nmol of TNB/mg of protein.

#### 4.10.4. Antioxidative Defenses

Reduced glutathione (GSH) levels were measured using a standard method [39]. Briefly, tissues were homogenized in the presence of 300 μL of 20 mmol/L sodium phosphate and 140 mmol/L KCl buffer, pH 7.4. Samples were centrifuged, and fifteen microliters of the tissue preparation was incubated with an equal volume of ophthaldialdehyde (1 mg/mL methanol) at room temperature for 15 min in the presence of 20 volumes (1:20, v/v) of 100 mmol/L sodium phosphate buffer, pH 8.0, containing 5 mM EDTA. Fluorescence was measured at wavelengths of 350 nm and 420 nm. GSH concentrations were calculated as nmol/mg protein.

#### 4.10.5. Glutathione Peroxide, Glutathione Reductase, and Superoxide Dismutase Activities

The glutathione peroxidase (GPx) assay was carried out as described in [40]. The measurement of enzymatic activity was performed by monitoring the loss of NADPH at 340 nm. The GPx (U) unit was defined as 1 μmol of NADPH consumed per minute. Glutathione reductase (GR) activity was performed as described [41]. Enzyme activity was determined by monitoring the NADPH consumption at 340 nm. Specific activity was calculated as U/mg of protein. Superoxide dismutase (SOD) activity was measured according to [42]. The absorbance was read at 420 nm.

#### 4.10.6. Activities of Glutamate Dehydrogenase (GDH), α-Ketoglutarate Dehydrogenase (α-KGDH), and Succinate Dehydrogenase (SDH)

The activities of GDH [43] and α-KGDH [44] were determined by a Spectramax M5 microplate spectrofluorometer using crude liver homogenates (GDH: 0.15 mg protein mL^−1^; α-KGDH: 0.25 mg protein mL^−1^). The activity of both enzymes was determined based on the reduction in NAD^+^ and expressed as nmol·min^−1^·mg protein^−1^. The SDH activity [45] was measured using the liver homogenate (0.15 mg protein mL^−1^) and the reduction in 2,6-dichloroindophenol (DCIP) at 600 nm; the data was expressed as nmol·min^−1^·mg protein^−1^.

### 4.11. Western Blot Analysis

Liver samples were prepared as described in [46]. Briefly, 20 μg of protein from each sample was fractionated by 8–12% SDS-PAGE and transferred to an immobilon PVDF membrane (Millipore, Billerica, MA, USA). The following primary antibodies were used: anti-D3 (1:1000; NovusBio, Englewood, CO, USA); anti-GRP78 (1:700; Invitrogen, Waltham, MA, USA); anti-HSPA9/GRP75 (1:500; Invitrogen) and anti-β-actin (1:25,000; Sigma-Aldrich, St. Louis, MO, USA). Antigen–antibody complexes were visualized using HRP-conjugated secondary antibodies and an enhanced chemiluminescence system (GE Healthcare, Pittsburgh, PA, USA). Expression was quantified using image densitometry with the ImageJ analysis software.

### 4.12. Statistical Analysis

Normality was verified for all variables using the Shapiro–Wilk test and histograms. Parametric data were analyzed using one/two-way analysis of variance followed by Tukey’s post hoc test, whereas nonparametric data were analyzed using the Kruskal–Wallis test. Longitudinal data analysis was performed using generalized estimating equations followed by Bonferroni’s post hoc test. Quantitative variables are expressed as the mean ± standard deviation or the median and interquartile range (25th–75th). Statistical significance was set at *p* < 0.05. Data were analyzed using Prism 10.0 software (Graphpad, San Diego, CA, USA).

## 5. Conclusions

To the best of our knowledge, this is the first preclinical study that evaluates the effect of LOLA on the Krebs cycle, redox state, and thyroid metabolism in MASLD, showing that this molecule considerably decreases or corrects the biochemical parameters of the serum lipid profile and the deposition of collagen fibers in the liver tissue. Most of all, even in the early stages, the energy production by the Krebs cycle is compromised, and enzymes are already reallocated to detoxify ammonia, which LOLA corrects in a very early stage of disease, as has just been demonstrated. These results add to the understanding of the physiopathology of MASLD and assess its translatability to a preclinical model for human steatohepatitis, helping to bridge the gap between preclinical and clinical studies.

## Figures and Tables

**Table 1:** Biochemical parameters, inflammatory gene expression, and vascular adhesion protein-1 level.

Biochemical Parameters					
Variables ^#^	Control	MASLD	LOLA	VitE	*p* *
AST (U/L)	76.14 ^a^ (±12.0)	142.1 ^b^ (±68.7)	108.5 ^b^ (±36.0)	105.5 ^b^ (±33.5)	<0.05
ALT (U/L)	39.4 (±7.5) ^a^	79.9 (±16.0) ^b^	50.9 (±18.1) ^a^	54.0 (±12.3) ^a^	<0.01
Glucose (mg/dL)	121.3 (±7.4) ^a^	147.3 (±21.5) ^b^	150.6 (±15.3) ^b^	144.6 (±23.7) ^b^	<0.05
Total cholesterol (mg/dL)	90.3 (±7.9) ^a^	131.4 (±15.0) ^b^	108.9 (±51.5) ^a,b^	102.9 (±29.1) ^a,b^	0.03
LDL cholesterol (mg/dL)	50.4 (±15.2) ^a^	86.9 (±12.6) ^b^	71.8 (±31.2) ^a,b^	74.8 (±24.7) ^a,b^	0.009
HDL cholesterol (mg/dL)	27.3 (±5.4) ^a^	17.2 (±5.6) ^b^	17.3 (±4.4) ^b^	18.3 (±4.1) ^b^	<0.001
Triglycerides (mg/dL)	75.9 (±22.0) ^a^	120.9 (±20.1) ^b^	60.33 (±21.7) ^a^	64.7 (±26.9) ^a^	<0.005
**Gene expression of hepatic inflammatory markers**					
*Il1b mRNA* (Fold)	1 (±0.59) ^a^	4.58 (±3.35) ^a,b^	3.21 (±2.44) ^a^	15.71 (±18.11) ^b^	<0.05
*Il6 mRNA* (Fold)	1 (±0.49) ^a^	8.78 (±4.31) ^b^	3.46 (±3.66) ^a,b^	10.32 (±10.19) ^b^	<0.05
*Il10 mRNA* (Fold)	1 (±0.08) ^a^	0.30 (±0.23) ^a,b^	1.23 (±1.20)	0.17 (±0.15) ^b^	<0.05
*TNF-α mRNA* (Fold)	1 (±0.76)	2.07 (±3.59)	1.45 (±1.39)	0.68 (±0.79)	0.996
**Vascular Adhesion Protein-1**					
(ng/mL)	2.05 (±1.25) ^a^	4.66 (±1.10) ^b^	3.92 (±0.84) ^b^	5.03 (±1.98) ^b^	<0.05

Notes: ^#^ Variables are described as mean ± standard deviation; * ANOVA test; *p* < 0.05 is considered significant. ^a^ compares the control and the MASLD group, whereas ^b^ compares the MASLD group with the other intervention groups when statistically significant. Abbreviations: AST, aspartate aminotransferase; ALT, alanine aminotransferase; LDL, low-density lipoprotein; HDL, high-density lipoprotein. *Il1b*, interleukin-1b; *Il6*, interleukin-6; *Il10*, interleukin-10; *TNF-α*, tumor necrosis factor-α.

**Table 2:** Primers used.

Gene	Forward	Reverse
*Il1b*	CAGAACATAAGCCAACAAGTGGTATT	CACAGGGATTTTGTCGTTGCT
*Il6*	TCCTACCCCAACTTCCAATGCTC	TTGGATGGTCTTGGTCCTTAGCC
*Il10*	GAGAGAAGCTGAAGACCCTCT	TCATTCATGGCCTTGTAGACAC
*Tnfa*	AAATGGGCTCCCTCTCATCAGTTC	TTGGATGGTCTTGGTCCTTAGCC
*Dio1*	ATTTGACCAGTTCAAGAGACTCGTAG	GGCGTGAGCTTCTTCAATGTA
*Dio3*	AACAGGGTGAAAGAGGGACATGGT	TAATCCCTTCTCCAAGGAGTCCTAGCCT
*Chop*	CCAGCAGAGGTCACAAGCAC	CGCACTGACCACTCTTGTTTC
*Bcl2*	GGCATCTGCACACCTGGAT	GGGCCATATAGTTCCACAAAGG
*Ucp2*	TCAACTGTACTGAGCTGGTGACCTA	GGAGGTCGTCTGTCATGAGGTT
*Pgc1a*	GGAGCAATAAAGCAAAGAGCA	GTGTGAGGAGGGTCATCGTT
*Gapdh*	CTACCCCCAATGTATCCGTTGT	ATGTCATCATACTTGGCAGGTTTC
*CyPA*	GTCAACCCCACCGTGTTCTTC	ACTTGCCACCAGTGCCATTATG

## Data Availability

The data presented in this study are available on request from the corresponding author. They are not publicly available because some data are still unpublished.
